# Tanshinone IIA Modulates Low Density Lipoprotein Uptake via Down-Regulation of PCSK9 Gene Expression in HepG2 Cells

**DOI:** 10.1371/journal.pone.0162414

**Published:** 2016-09-12

**Authors:** Hung-Chen Chen, Pei-Yi Chen, Ming-Jiuan Wu, Mi-Hsueh Tai, Jui-Hung Yen

**Affiliations:** 1 Department of Molecular Biology and Human Genetics, Tzu Chi University, Hualien, Taiwan; 2 Center of Medical Genetics, Buddhist Tzu Chi General Hospital, Hualien, Taiwan; 3 Department of Biotechnology, Chia-Nan University of Pharmacy and Science, Tainan, Taiwan; Centro Cardiologico Monzino, ITALY

## Abstract

Tanshinone IIA, one of the most pharmacologically bioactive phytochemicals isolated from *Salvia miltiorrhiza* Bunge, possesses several biological activities such as anti-inflammation, anti-cancer, neuroprotection and hypolipidemic activities. In this study, we aim to investigate the hypocholesterolemic effect of tanshinone IIA in hepatic cells. We demonstrated that tanshinone IIA significantly increased the amount of low-density lipoprotein receptor (LDLR) and LDL uptake activity in HepG2 cells at the post-transcriptional regulation. We further demonstrated that tanshinone IIA inhibited the expression of proprotein convertase subtilisin/kexin type 9 (PCSK9) mRNA and mature protein, which may lead to an increase the cell-surface LDLR in hepatic cells. We further identified a regulatory DNA element involved in the tanshinone IIA-mediated PCSK9 down-regulation, which is located between the -411 and -336 positions of the PCSK9 promoter. Moreover, we found that tanshinone IIA markedly increased the nuclear forkhead box O3a (FoxO3a) level, enhanced FoxO3a/PCSK9 promoter complexes formation and decreased the PCSK9 promoter binding capacity of hepatocyte nuclear factor 1α (HNF-1α), resulting in suppression of PCSK9 gene expression. Finally, we found that the statin-induced PCSK9 overexpression was attenuated and the LDLR activity was elevated in a synergic manner by combination of tanshinone IIA treatment in HepG2 cells. Overall, our results reveal that the tanshinone IIA modulates LDLR level and activity via down-regulation of PCSK9 expression in hepatic cells. Our current findings provide a molecular basis of tanshinone IIA to develop PCSK9 inhibitors for cholesterol management.

## Introduction

Epidemiological studies have demonstrated that elevated circulating triglyceride-rich remnant lipoproteins and high concentrations of plasma low-density lipoprotein cholesterol (LDL-C) are directly correlated with the risk of atherosclerosis and cardiovascular disorders [1, 2]. The circulating LDL level is primarily determined by its rate of uptake through the LDL receptor (LDLR), a membrane glycoprotein in the human liver. Hepatic LDLR mediates the internalization of receptor-bound LDL particles and delivers the complex to the endosomes for degradation, and the LDLR then returns to the cell membrane. The free cholesterol is further metabolized to bile acids for excretion in hepatic cells [3]. Thus, the hepatic expression of LDLR plays a critical role in removing plasma LDL and is important in modulating cholesterol homeostasis. Raising the level of the cell-surface LDLR and receptor activity to increase the cholesterol clearance is an efficient way to reduce LDL levels in the bloodstream [4].

The level of LDLR is modulated in cells at transcriptional and post-transcriptional regulation [5]. LDLR transcription is predominantly activated by the transcription factors sterol response element binding proteins (SREBPs), especially nuclear SREBP1 or SREBP2 proteins, which bind to sterol regulatory elements (SREs) within the LDLR promoter [6]. In addition to the transcriptional regulation of LDLR, the proprotein convertase subtilisin/kexin type 9 (PCSK9) is an important post-transcriptional regulator for modulating the amount of LDLR protein. The PCSK9 protein is translated as an approximately 74 kDa precursor protein and is processed into a 60 kDa secreted mature protein [7]. The secreted PCSK9 protein is an identified subtilisin-related serine protease that binds LDLR, blocks LDLR recycling and promotes LDLR degradation in the lysosome [8]. Thus, PCSK9 expression controls the LDLR level and cholesterol homeostasis. Individuals with loss-of-function PCSK9 mutations exhibit reductions in LDL-C levels and a risk of coronary heart disorders [9]. In recent clinical trials, monoclonal antibodies against PCSK9 caused a decrease in the plasma LDL-C levels of patients with hypercholesterolemia [10]. The inhibition of PCSK9 function might be expected to serve as a novel approach to modulate hepatic LDLR levels and manage lipid metabolism [11, 12].

PCSK9 gene expression was regulated by several transcription factors such as SREBPs, hepatocyte nuclear factor 1α (HNF-1α) and forkhead box O3 (FoxO3) [13–15]. Activation of the SREBP pathway has been reported to increase both LDLR and PCSK9 transcriptional activities. PCSK9, as well as LDLR, contain SREs in their promoters that respond to alterations in cellular cholesterol levels through SREBP-dependent pathways. The PCSK9 promoter interacts with SREBP2 or SREBP1c, which stimulates PCSK9 gene expression [16]. The transcription factor HNF-1α was also found to bind a DNA element within the PCSK9 promoter and to activate PCSK9 expression in hepatic cells. The attenuation of nuclear HNF-1α levels resulted in the down-regulation of PCSK9 expression, consequently increased the amount of cell-surface LDLR and elevated the receptor activity in hepatic cell lines [14, 17]. Recently, the FoxO3a protein, a forkhead transcription factor, has been reported as a negative regulator of PCSK9 gene expression through epigenetic modulation. The FoxO3a protein interacts with the insulin-response element (IRE) within the PCSK9 promoter, recruiting the sirtuin-6 (Sirt6) protein to deacetylate histones and reducing the promoter binding capacity of HNF-1α, which leads to a suppression of PCSK9 gene expression in hepatic cells [15].

**Fig 1:**
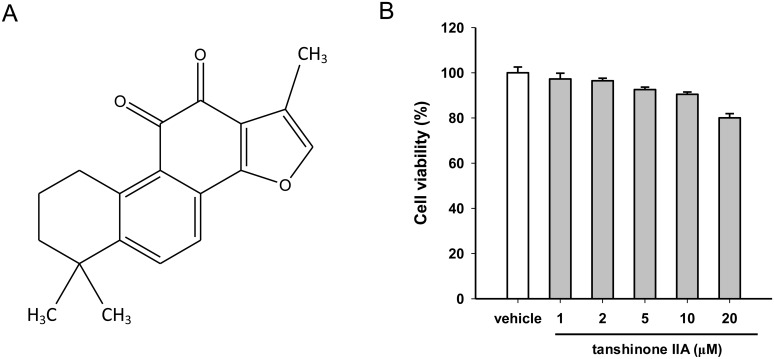
Effect of tanshinone IIA on the cell viability in HepG2 cells. (A) Chemical structure of tanshinone IIA. (B) HepG2 cells were cultured in LPDS medium and treated with the vehicle (0.1% DMSO) or tanshinone IIA (1–20 μM) for 24 h. Cell viability was measured using an MTT assay. The data represent the mean ± SD from three independent experiments.

The dried root of *Salvia miltiorrhiza* Bunge, also known as Danshen, is a traditional Chinese herbal medicine that has been used in Asian countries for the treatment of coronary artery disease, myocardial infarction, stroke, atherosclerosis and cerebrovascular disorders [18, 19]. Tanshinone IIA (Fig 1A) is one of the most pharmacologically bioactive constituents isolated from Danshen and possesses numerous biological activities for the prevention or treatment of inflammation processes, hepatic fibrosis, neurodegenerative disorders, cancer and cardiovascular diseases [20, 21]. Several studies have suggested that tanshinone IIA modulates lipid metabolism. In animal studies, it has been demonstrated that tanshinone IIA attenuated atherosclerosis in hypercholesterolemic rats [22] and hyperlipidemic rabbits [23]. Tanshinone IIA prevented foam cell formation and oxidized-LDL (oxLDL) uptake in macrophages [24], reduced oxLDL-induced scavenger receptor expression through activation of the ERK/Nrf2/HO-1 pathway and regulated the cholesterol balance [25]. However, the underlying mechanisms of tanshinone IIA’s regulation of cholesterol homeostasis remain unclear.

In the present study, we aim to investigate the hypocholesterolemic effects of tanshinone IIA and underlying mechanisms in HepG2 cells, which are generally used as a model to study the human LDL-C metabolism [26]. We investigated the effect of tanshinone IIA on the LDLR level, LDL uptake activity and PCSK9 gene expression in HepG2 cells. We also focus on the effects of combining tanshinone IIA and lovastatin or simvastatin on PCSK9 gene expression and LDLR activity in HepG2 cells.

## Materials and Methods

### Chemicals

Tanshinone IIA (purity≧ 97%), dimethyl sulfoxide (DMSO), non-essential amino acids (NEAA), mevinolin (lovastatin) and other chemicals were purchased from Sigma-Aldrich Co. (St. Louis, MO), unless otherwise indicated. Gibco^®^ fetal bovine serum (FBS) was purchased from Thermo Fisher Scientific, Inc. (Rockford, IL, USA). Simvastatin was purchased from Tokyo Chemical Industry (Toshima, Kita-Ku, Tokyo, Japan).

### Cell culture and treatment of cells with tanshinone IIA

HepG2 cells were obtained from the Bioresource Collection and Research Center (Hsinchu, Taiwan) and maintained in DMEM medium containing 10% fetal bovine serum (FBS). The cells were seeded and cultured in normal serum medium overnight; then, the medium was changed to DMEM supplemented with 5% lipoprotein-deficient serum (LPDS) [27] and was cultured for 24 h. The cells were then treated with vehicle (0.1% DMSO) or tanshinone IIA for 24 h. For statin treatment, the HepG2 cells were pretreated with the vehicle or tanshinone IIA for 1 h and then incubated with 1 μM lovastatin or simvastatin for an additional 24 h.

### MTT assay

The MTT assay was used to determine the cell viability [28]. The cells were treated with the vehicle or tanshinone IIA (1–20 μM) for 24 h and then incubated with MTT reagent (1 mg/mL) at 37°C for 3 h. The purple formazan crystals were dissolved in DMSO and the absorbance was measured at 550 nm.

### RNA extraction and reverse transcription quantitative PCR (RT-Q-PCR) analysis

Cells were cultured in LPDS-containing medium as described above and treated with the vehicle or tanshinone IIA (5 and 10 μM) for 24 h. The RNA was extracted using the Total RNA mini Kit (Geneaid, Taipei, Taiwan) according to the manufacturer’s instructions. Reverse transcription was carried out using the High-Capacity cDNA reverse transcription kit (Thermo Fisher Scientific). Quantitative real-time PCR was performed using a reaction mixture containing cDNA, specific primers [LDLR, 5'-AGTTGGCTGCGTTAATGTGA-3' (forward) and 5'-TGATGGGTTCATCTGACCAGT-3' (reverse); PCSK9, 5'-GCTGAGCTGCTCCAGTTTCT -3' (forward) and 5'-AATGGCGTAGACACCCTCAC-3' (reverse); HMG-CoA reductase (HMGCR), 5'- TGATTGACCTTTCCAGAGCAAG-3' (forward) and 5'- CTAAAATTGCCATTCCACGAGC-3' (reverse)]; GAPDH, 5'- CATGAGAAGTATGACAACAGCCT-3' (forward) and 5'- AGTCCTTCCACGATACCAAAGT-3' (reverse)] [17] and Maxima SYBR Green/ROX qPCR Master Mix (Thermo Fisher Scientific). PCR amplification was carried out in a Roche LightCycler^®^-480 Real-Time PCR System. The real-time PCR conditions were 50°C for 2 min, 94°C for 4 min, 45 cycles of 94°C for 1 min, 58°C for 1 min, and 72°C for 1 min. The ΔΔC_t_ method was used for data analysis [29]. The amount of mRNA was normalized to the GAPDH level in the same samples.

### Western blot analysis

Cells were seeded as described above, the medium was then changed to a DMEM/LPDS medium and the vehicle or tanshinone IIA was added for an additional 24 h. For the extraction of total cellular proteins, the cells were harvested using RIPA buffer (Thermo Fisher Scientific). For nuclear extract preparation, the cells were harvested using the NE-PER nuclear and cytoplasmic extraction reagent (Thermo Fisher Scientific) according to the manufacturer’s instructions. Total cellular lysates (20 μg) and nuclear extracts (20 μg) were analyzed by 10% SDS-PAGE and transferred onto a PVDF membrane (PerkinElmer, Boston, MA, USA). The blots were incubated with the following specific primary antibodies at 4°C for 24 h: anti-LDLR (Novus Biologicals, Littleton, CO, USA), anti-PCSK9, anti-HDAC2 (GeneTex, Irvine, CA, USA), anti-N-terminus of SREBP2 (Cayman, Ann Arbor, MI, USA), anti-HNF-1α (Santa Cruz Biotechnology, Santa Cruz, CA, USA), anti-FoxO3a (Cell Signaling Technology, Danvers, MA) or anti-β-actin (Sigma-Aldrich). The blots were incubated with the appropriate horseradish peroxidase-conjugated secondary antibodies (Santa Cruz Biotechnology) at 37°C for 1 h. The proteins were detected using the Amersham ECL^™^ Prime Western Blotting Detection Reagent, and the signal was visualized on Amersham Hyperfilm^™^ ECL (GE Healthcare, Buckinghamshire, UK).

### Analysis of cell-surface LDLR

The cell-surface LDLR was detected by flow cytometry analysis as previously described [17]. Briefly, the cells were seeded as described above and treated with the vehicle or tanshinone IIA for 24 h. The cells were scraped, washed with phosphate-buffered saline (PBS) and then incubated with PBS containing 5% bovine serum albumin (BSA) at room temperature for 30 min. The anti-LDLR antibody was added and incubated at 37°C for 1 h. The cells were washed with PBS and incubated with Alexa Fluor 488-conjugated goat anti-rabbit IgG (Thermo Fisher Scientific) at room temperature for 30 min. The cells were analyzed by flow cytometry (FACScan, BD Biosciences, San Jose, CA, USA). The data were calculated using Cell Quest Pro software (BD Biosciences), and the cell-surface LDLR was expressed as the relative percentage of the geometric mean fluorescence intensity.

### Measurement of LDL uptake

The amount of LDL uptake was measured as previously described [17, 30]. Briefly, the cells were treated with vehicle or tanshinone IIA for 24 h, which was then replaced with fresh serum-free medium and incubated with 10 μg/mL BODIPY^®^-FL-LDL (Thermo Fisher Scientific) at 37°C for 24 h. The cells were detached, washed and re-suspended in PBS and examined by flow cytometry. Data were acquired from 10,000 cells (counts). The LDL uptake data were expressed as the relative percentage of the geometric mean fluorescence intensity.

### Plasmid transfection and luciferase reporter assay

Reporter plasmids containing the PCSK9 promoter (from −1459 to −4, relative to the A of the translation initiation codon ATG), the LDLR promoter (from −1480 to +40, relative to the transcription start site), and the serial deletion regions of the PCSK9 promoter for the luciferase reporter assay were designated and constructed as previously described [17]. HepG2 cells were co-transfected with the indicated reporter plasmids and the *Renilla* luciferase control vector (Promega) using the Lipofectamine^®^ regent (Thermo Fisher Scientific) according to the manufacturer’s instructions. After transfection, the cells were treated with vehicle or tanshinone IIA for 24 h. The luciferase activities were measured using the Dual-Luciferase Reporter Assay System Kit (Promega), and the intensities were normalized to the *Renilla* luciferase activity.

### Chromatin immunoprecipitation (ChIP) assay

The ChIP assay was performed using the ChIP Assay Kit (Millipore, Billerica, MA, USA) as previously described [17, 31]. Briefly, the chromatin isolated from cells was sheared by sonication, and 1% of the sheared products were as an input control. The chromatin preparation was incubated with specific antibodies including anti-FoxO3a, anti-HNF-1α or control IgG (Millipore) at 4°C for 24 h. Quantitative real-time PCR was carried out to enrich the promoter binding levels determined using the PCSK9 ChIP primers 5'-TCCAGCCCAGTTAGGATTTG-3' and 5'-CGGAAACCTTCTAGGGTGTG-3'[14, 17]. The data were analyzed by ΔΔC_t_ method [32] and expressed as fold enrichment (fold increase compared to the control IgG).

### Statistical Analysis

All experiments were replicated at least three times, and the values are expressed as the mean ± SD. The results were analyzed using one-way ANOVA with Dunnet’s post hoc test, and a *p* value < 0.05 was considered statistically significant.

## Results

### Effect of tanshinone IIA on the LDLR expression and LDLR activity

**Fig 2:**
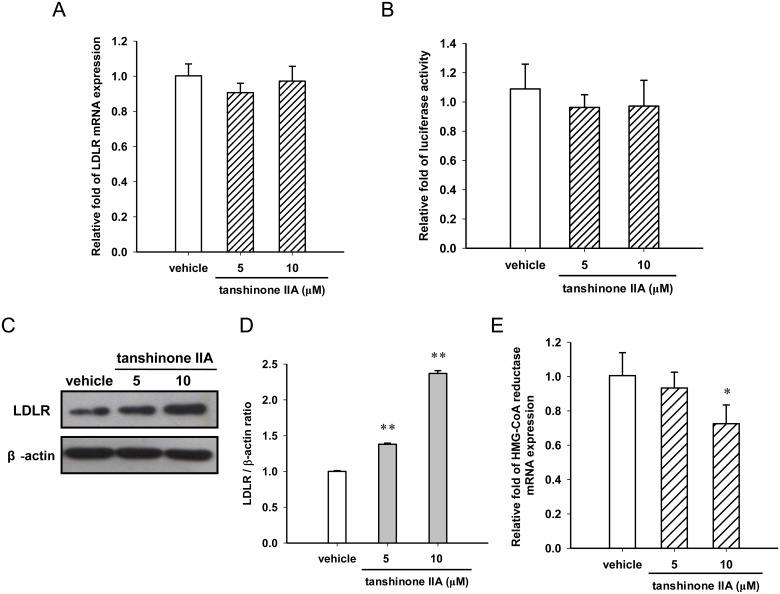
Effect of tanshinone IIA on the LDLR mRNA and protein expression. (A) HepG2 cells were cultured in LPDS medium and treated with the vehicle (0.1% DMSO) or tanshinone IIA (5 and 10 μM) for 24 h. The LDLR mRNA was measured by RT-Q-PCR. (B) The LDLR promoter-luciferase reporter construct and a *Renilla* luciferase control plasmid were co-transfected into HepG2 cells for 24 h, and the cells were then treated with the vehicle (0.1% DMSO) or tanshinone IIA (5 and 10 μM) for 24 h. The luciferase activities were measured, and the data represent the mean ± SD from three independent experiments. (C) The level of LDLR protein was determined by western blot analysis. A representative blot is shown. (D) The normalized intensity of LDLR versus β-actin is presented as the mean ± SD of three independent experiments. (E) The HMG-CoA reductase mRNA was measured by RT-Q-PCR. *p<0.05 and **p<0.01 represent significant differences compared to the vehicle group.

First, the cytotoxic effect of tanshinone IIA on HepG2 cells was examined. The cells were cultured in lipoprotein-deficient serum (LPDS)-containing medium and treated with vehicle (0.1% DMSO) or tanshinone IIA (1–20 μM) for 24 h. The cell viability was determined by MTT assay. As shown in Fig 1B, tanshinone IIA (1–20 μM) exerted no significant cytotoxicity on HepG2 cells. We further investigated the effect of tanshinone IIA on the LDLR gene expression. As shown in Fig 2A and 2B, tanshinone IIA (5 and 10 μM) did not significantly change the LDLR mRNA expression or the transcriptional activity of the LDLR promoter in HepG2 cells. Furthermore, the effect of tanshinone IIA on LDLR protein expression were analyzed in HepG2 cells. Western blot analysis showed that the cellular LDLR proteins were significantly increased in the tanshinone IIA (5 and 10 μM)-treated cells compared to the vehicle control group (*p*<0.01) (Fig 2C and 2D). Moreover, the effect of tanshinone IIA on HMG-CoA reductase (HMGCR) expression, a key enzyme for cholesterol biosynthesis, was also examined in HepG2 cells. As shown in Fig 2E, tanshinone IIA at concentration of 10 μM slightly decreased the HMGCR mRNA expression, however, cells treated with 5 μM tanshinone IIA expressed similar levels of HMGCR. These data indicate that tanshinone IIA modulates the LDLR expression may not be the result on the transcriptional regulation or inhibition of cholesterol biosynthesis.

**Fig 3:**
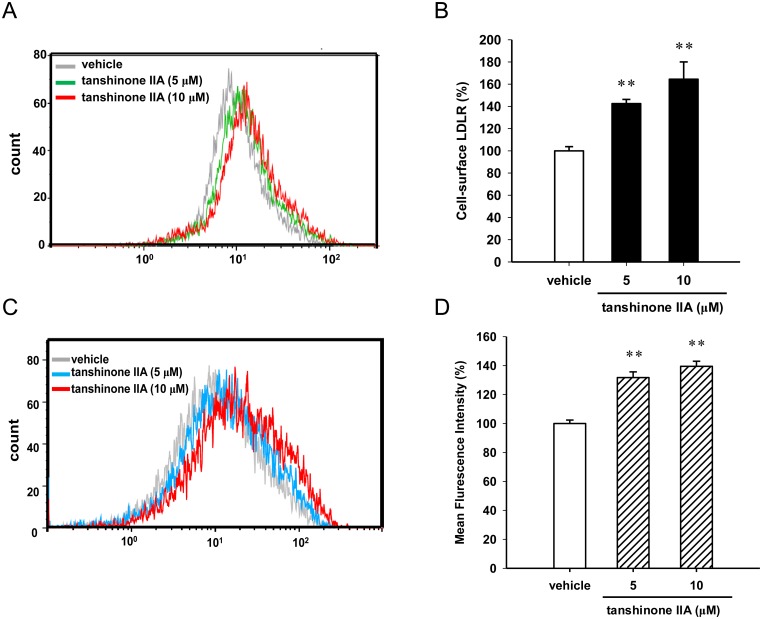
Effect of tanshinone IIA on the cell-surface LDLR levels and LDL uptake in HepG2 cells. HepG2 cells were cultured in LPDS medium and treated with the vehicle (0.1% DMSO) or tanshinone IIA (5 and 10 μM) for 24 h. (A) The level of cell-surface LDLR was determined by flow cytometry analysis. A representative histogram is shown. (B) Summary of cell-surface LDLR levels. (C) BODIPY^®^-FL-LDL uptake was determined by flow cytometry analysis. A representative histogram is shown. (D) Summary of LDL uptake. The data represent the mean ± SD from three independent experiments. ** *p*<0.01 represent significant differences compared to the vehicle group.

Furthermore, the effect of tanshinone IIA on the level of cell-surface LDLR was investigated using flow cytometry analysis, as described in Materials and Methods section. As shown in Fig 3A and 3B, the cell-surface LDLR level on the HepG2 cells was significantly increased by approximately 40% and 60% in the tanshinone IIA (5 and 10 μM)-treated cells, respectively. The mean fluorescence intensity results showed that tanshinone IIA (5 and 10 μM)-treated cells markedly enhanced the LDL uptake by approximately 30% and 40% compared to the vehicle control (*p*<0.01), respectively (Fig 3C and 3D). These above results suggest that tanshinone IIA increases the amount of LDLR protein at the post-transcriptional level and enhances the LDL uptake activity in the HepG2 cells.

### Tanshinone IIA down-regulates the PCSK9 gene expression in HepG2 cells

**Fig 4:**
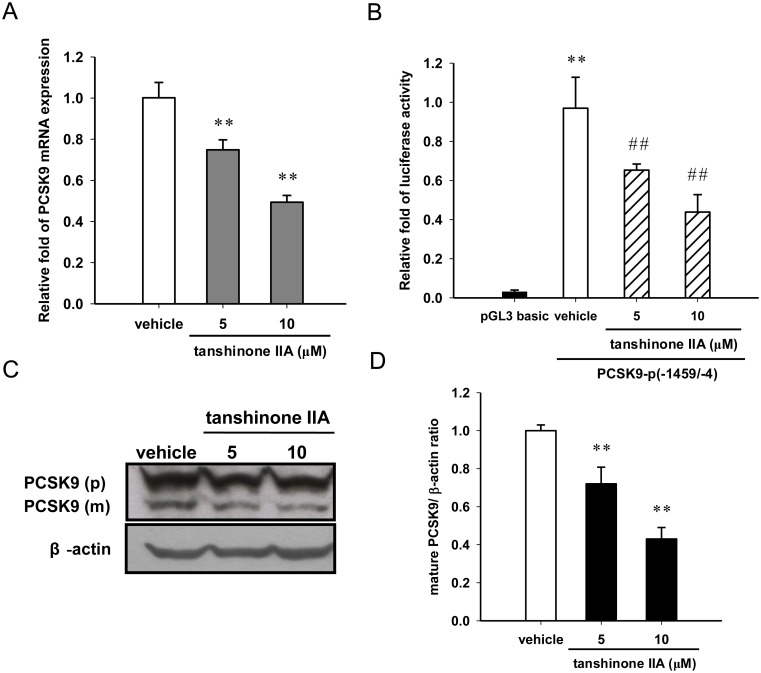
Effect of tanshinone IIA on PCSK9 mRNA and protein expression. (A) HepG2 cells were cultured in LPDS medium and treated with the vehicle (0.1% DMSO) or tanshinone IIA (5 and 10 μM) for 24 h. PCSK9 mRNA expression was measured by RT-Q-PCR. The data represent the mean ± SD from three independent experiments. ** *p*<0.01 represent significant differences compared to the vehicle-treated cells. (B) The cells were transfected with a pGL3-basic plasmid (as a vector control) or PCSK9 promoter reporter construct (PCSK9-p(-1459/-4)) and a *Renilla* control plasmid for 24 h. The cells were then treated with the vehicle (0.1% DMSO) or tanshinone IIA (5 and 10 μM) for 24 h. The luciferase activities were measured, and the data represent the mean ± SD from three independent experiments. ** *p*<0.01 represent significant differences compared to the pGL3-basic vector-transfected cells. ## *p*<0.01 represent significant differences compared to the vehicle-treated cells. (C) PCSK9 precursor (p) and mature form (m) were measured by western blot analysis. A representative blot is shown. (D) The normalized intensity of mature PCSK9 (m) versus β-actin is presented as the mean ± SD of three independent experiments. ** *p*<0.01 represent significant differences compared to the vehicle-treated cells.

PCSK9 protein is a known regulator for post-transcriptional modulation of LDLR in hepatic cells. Therefore, the effect of tanshinone IIA on PCSK9 gene expression was further investigated in HepG2 cells. As shown in Fig 4A, HepG2 cells treated with 5 and 10 μM tanshinone IIA significantly inhibited the PCSK9 mRNA expression by approximately 24% and 51% compared to the vehicle-treated group (*p*<0.01), respectively. We further examined the effect of tanshinone IIA on the transcriptional activity of PCSK9 promoter by transfecting cells with a PCSK9 promoter reporter plasmid (PCSK9-p(-1459 /-4)) and a *Renilla* luciferase control plasmid, as described in Materials and Methods section. The result showed that the PCSK9 promoter activity was significantly reduced in the 5 and 10 μM tanshinone IIA-treated cells compared to the vehicle-treated cells (*p*<0.01) (Fig 4B). Furthermore, we examined the PCSK9 protein expression in tanshinone IIA-treated cells by western blot analysis. As shown in Fig 4C and 4D, tanshinone IIA significantly reduced the level of mature PCSK9 protein in HepG2 cells. These results indicate that tanshinone IIA-mediated PCSK9 down-regulation through the inhibition of the PCSK9 promoter activity.

### Characterization of tanshinone IIA-responsive element upon PCSK9 promoter

**Fig 5:**
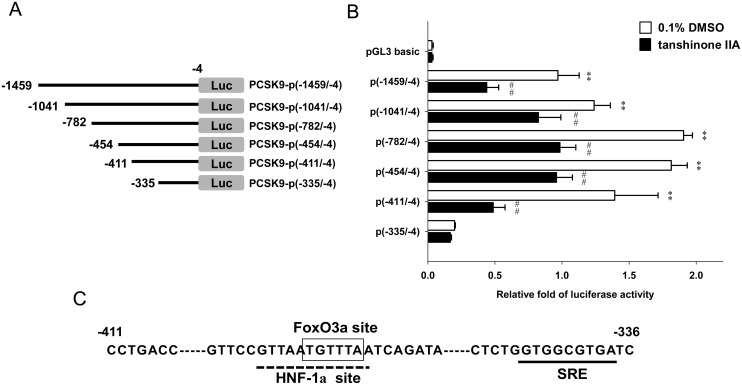
Effect of tanshinone IIA on PCSK9 promoter activity. (A) Serially deleted PCSK9 promoter reporter constructs. (B) HepG2 cells were transfected with the serial deleted PCSK9 promoter reporter constructs for 24 h and then treated with the vehicle (0.1% DMSO) or tanshinone IIA (10 μM) for 24 h. The luciferase activities were determined and normalized to their respective *Renilla* luciferase control activities. The data represent the mean ± SD from three independent experiments. **p<0.01 represents a significant difference compared to the pGL3-basic vector-transfected cells. ## *p*<0.01 represents a significant difference compared to the respective vehicle-treated group. (C) The analysis of the tanshinone IIA-response element within the PCSK9 promoter. The previously characterized HNF-1α, SREBP2 and FoxO3a binding sites are indicated as HNF-1α site, SRE and FoxO3a site, respectively.

To investigate the molecular mechanism underlying the tanshinone IIA-mediated PCSK9 down-regulation, the regulatory DNA elements of the PCSK9 promoter that are responsive to the tanshinone IIA-mediated PCSK9 reduction were further identified. HepG2 cells were transfected with reporter plasmids containing serial deletions of the PCSK9 promoter [17] (Fig 5A) followed by exposure with vehicle or tanshinone IIA (10 μM) for 24 h. As shown in Fig 5B, the luciferase activities of tanshinone IIA-treated cells were significantly decreased as compared to the vehicle-treated cells (*p*<0.01). Additionally, we found that the PCSK9 promoter activity was strongly attenuated in those cells transfected with the reporter plasmid PCSK9-p(-335/-4). These data indicate that the DNA region located between the -411 and -336 positions on PCSK9 proximal promoter is a critical regulatory element for tanshinone IIA-mediated PCSK9 down-regulation. It has been reported that this region of the PCSK9 promoter contains an HNF-1α binding site, a SREBP2 binding element (sterol regulatory element; SRE), as well as a FoxO3a binding site [14, 15, 33] (Fig 5C).

### Effects of tanshinone IIA on the nuclear HNF-1α, SREBP2 and FoxO3a proteins

**Fig 6:**
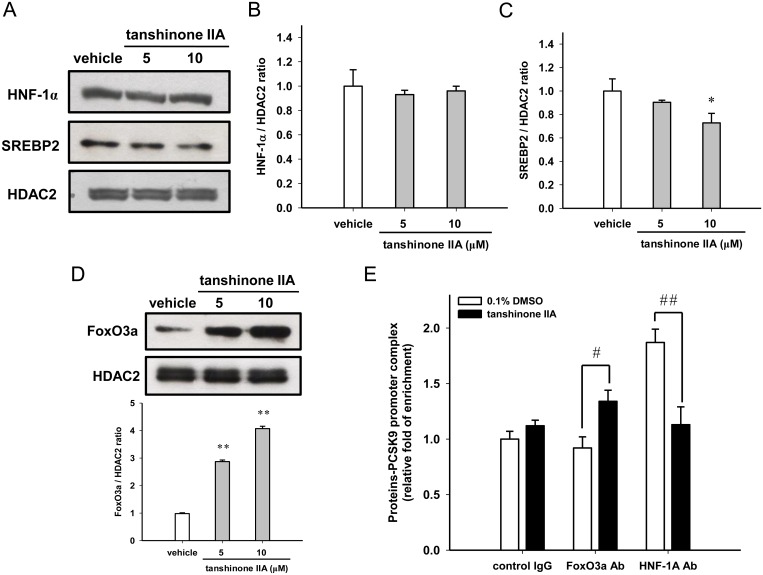
Effect of tanshinone IIA on the nuclear accumulation of HNF-1α, SREBP2 and FoxO3a proteins. (A) Levels of nuclear HNF-1α and SREBP2 proteins measured by Western blot analysis. A representative blot is shown. The normalized intensities of HNF-1α (B) and SREBP2 (C) versus HDAC2 are presented as the mean ± SD of three independent experiments. *p<0.05 represents a significant difference compared to the vehicle-treated group. (D) The nuclear FoxO3a protein was detected by western blot analysis. A representative blot is shown. The normalized intensity of FoxO3a versus HDAC2 is presented as the mean ± SD of three independent experiments. ** *p*<0.01 represents a significant difference compared to the vehicle-treated group. (E) The ChIP assay was performed as described in the Materials and Methods section. The PCSK9 promoter complex was measured by RT-Q-PCR. The data were expressed as the fold enrichment of the FoxO3a antibody (FoxO3a Ab) or HNF-1α antibody (HNF-1A Ab) over the control IgG. The data represent the mean ± SD from three independent experiments. # *p*<0.05 and ## *p*<0.01 represents a significant difference compared to the vehicle-treated control IgG group.

To investigate whether these transcription factors are involved in the tanshinone IIA-mediated PCSK9 down-regulation, the effect of tanshinone IIA on the levels of nuclear HNF-1α, SREBP2 and FoxO3a proteins were evaluated in HepG2 cells. As shown in Fig 6A–6C, when the cells were treated with 5 or 10 μM tanshinone IIA, the nuclear HNF-1α level was not significantly altered in the HepG2 cells. However, tanshinone IIA (10 μM) slightly reduced the nuclear SREBP2 protein in HepG2 cells. We further detected the nuclear accumulation of FoxO3a in 5 or 10 μM tanshinone IIA-treated HepG2 cells. The level of nuclear FoxO3a protein was significantly increased by 2.9- and 4.0-fold compared to the vehicle-treated cells, respectively (Fig 6D). Furthermore, the effect of tanshinone IIA on the *in vivo* interaction of FoxO3a with the PCSK9 promoter in HepG2 cells was examined using a ChIP assay, as described in Materials and Methods section. As shown in Fig 6E, cells were treated with tanshinone IIA (10 μM) significantly increased the FoxO3a/PCSK9 promoter complexes formation compared to the vehicle-treated cells. Additionally, we examined the effect of tanshinone IIA on the interaction of HNF-1α and PCSK9 promoter. We found that the nuclear HNF-1α/PCSK9 promoter complexes formation was significantly attenuated in tanshinone IIA-treated cells. These above results suggest that tanshinone IIA increases the accumulation of nuclear FoxO3a protein and its interaction with the PCSK9 promoter, which may lead to reduce the promoter binding capacity of HNF-1α and down-regulate the PCSK9 gene expression in HepG2 cells.

### Combination of tanshinone IIA and statins treatment promotes more LDL uptake in HepG2 cells

**Fig 7:**
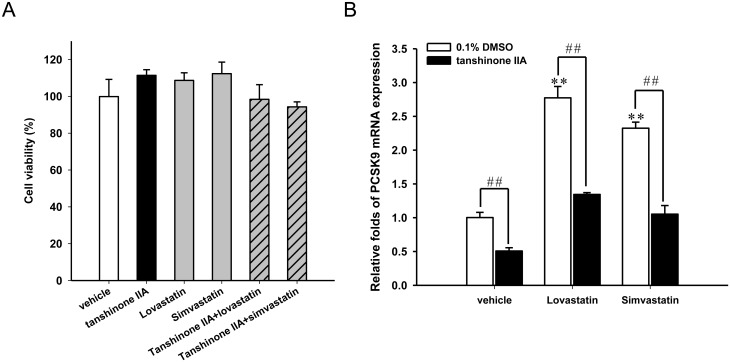
Effect of the combination of tanshinone IIA and statin treatments on PCSK9 mRNA expression. The HepG2 cells were cultured in LPDS medium and pretreated with the vehicle or tanshinone IIA (10 μM) for 1 h and then incubated with 1 μM lovastatin or simvastatin for an additional 24 h. (A) Cell viability was measured using an MTT assay. (B) The PCSK9 mRNA was measured by RT-Q-PCR. The data represent the mean ± SD from three independent experiments. **p<0.01 represents a significant difference compared to the vehicle-treated cells. ##*p*<0.01 represents a significant difference compared to the statin (lovastatin or simvastatin) alone treatment group.

**Fig 8:**
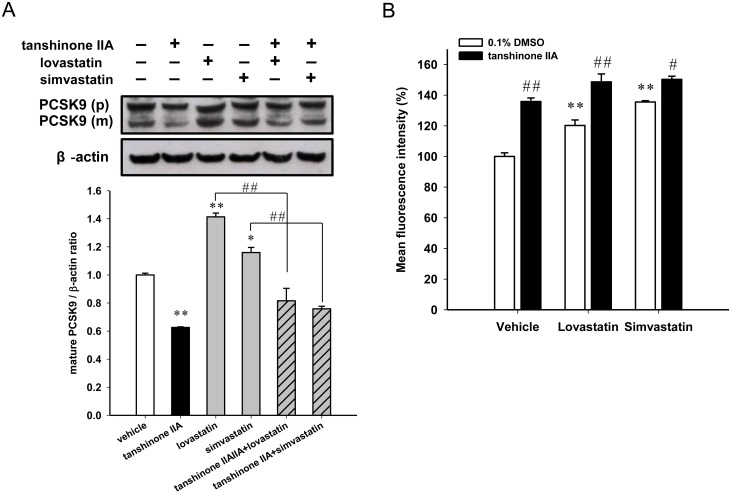
Effect of the combination of tanshinone IIA and statin treatments on PCSK9 protein expression and LDLR activity. The HepG2 cells were cultured in LPDS medium and pretreated with the vehicle or tanshinone IIA (10 μM) for 1 h and then incubated with 1 μM lovastatin or simvastatin for an additional 24 h. (A) The PCSK9 protein level was measured by western blot analysis. The immunoblot experiments were repeated three times, and a representative blot is shown. The normalized intensity of mature PCSK9 (m) versus β-actin is presented as the mean ± SD of three independent experiments. **p<0.01 represents a significant difference compared to the vehicle-treated cells. ##*p*<0.01 represents a significant difference compared to the statin (lovastatin or simvastatin) alone treatment group. (B) The LDL uptake by the cells was measured by flow cytometry analysis, and the data are expressed as the geometric mean fluorescence intensity. The data represent the mean ± SD from three independent experiments. **p<0.01 represents a significant difference compared to the tanshinone IIA-untreated vehicle group. ##*p*<0.01 represents a significant difference compared to the tanshinone IIA-untreated cells.

Statin treatment may increase the PCSK9 gene expression and attenuate the therapeutic ability for lowering the plasma LDL, thus, we further investigated whether a combination of tanshinone IIA and statins alter the PCSK9 expression and LDL uptake in HepG2 cells. The cytotoxic effect of a tanshinone IIA and statins combination was evaluated in HepG2 cells. As shown in Fig 7A, the combination of tanshinone IIA (10 μM) and lovastatin or simvastatin (1 μM) treatment showed no cytotoxicity on HepG2 cells. The cells treated with lovastatin or simvastatin alone significantly increased PCSK9 mRNA and mature protein expression (Figs 7B and 8A). The lovastatin- or simvastatin-induced PCSK9 expression were significantly attenuated by co-treatment with tanshinone IIA. As shown in Fig 8B, co-treatment of cells with tanshinone IIA and two statins significantly increased the LDL uptake activity as compared to cells treated with lovastatin or simvastatin alone. Our results reveal that a combination of tanshinone IIA and statins markedly attenuated the statin-induced PCSK9 gene expression and elevated LDLR activity in HepG2 cells.

## Discussion

**Fig 9:**
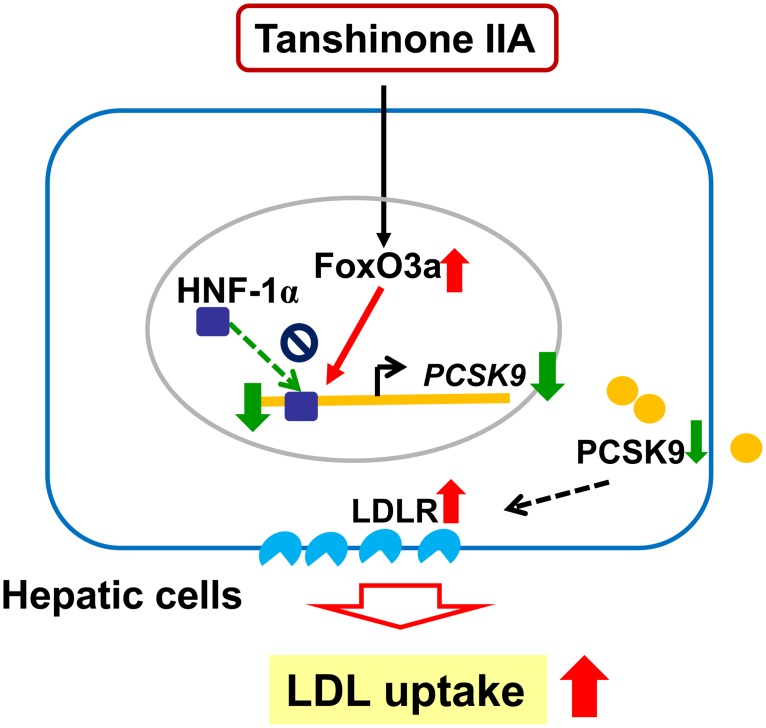
Hypothetical mechanism by which the tanshinone IIA increased LDL uptake via down-regulation of PCSK9 expression in hepatic cells. Tanshinone IIA increases the nuclear abundance of the FoxO3a and its interaction with PCSK9 promoter, which may result in the inhibition of HNF-1α/PCSK9 promoter complex formation and the decreases of PCSK9 gene expression. Down-regulation of PCSK9 gene expression by tanshinone IIA is associated with increases in the amount of cell-surface LDLR and its LDL-uptake activity in hepatic cells.

In the present study, we demonstrate that tanshinone IIA markedly down-regulates PCSK9 gene expression, which is associated with increases in the amount of cell-surface LDLR and its LDL-uptake activity in HepG2 cells. We show that tanshinone IIA dramatically elevates the nuclear abundance of the FoxO3a and its interaction with PCSK9 promoter, which may result in the inhibition of HNF-1α/PCSK9 promoter complex formation and the down-regulation of PCSK9 gene expression (Fig 9). Moreover, we demonstrate that tanshinone IIA attenuates the lovastatin- or simvastatin-induced PCSK9 expression and potentially combines treatment with statins.

The hepatic LDLR expression is involved in the regulation of plasma cholesterol homeostasis. It is known that patients with familial hypercholesterolemia, commonly mutations in the LDLR, experience high levels of LDL-C and premature atherosclerosis [34]. Elevated LDLR expression in hepatic cells results in increased clearance of plasma LDL through LDLR-mediated endocytosis and is associated with a decreased risk of cardiovascular disease [35, 36]. In the present study, we demonstrated that tanshinone IIA significantly increases the levels of total cellular and cell-surface LDLR protein and its LDL uptake activity in HepG2 cells. Our finding supported that tanshinone IIA possesses a cholesterol-lowering effect and has great potential for the prevention or treatment of hypercholesterolemia. The LDLR gene expression is transcriptionally activated by the sterol regulatory element binding proteins (SREBPs) [37]. We further investigated the effect of tanshinone IIA on the LDLR transcription in HepG2 cells. We found that the LDLR promoter activity and mRNA expression were not significantly altered by tanshinone IIA treatment in HepG2 cells. Our findings indicate that tanshinone IIA increases the level of hepatic LDLR protein at post-transcriptional regulation, not through transcriptional activation.

In addition to transcriptional control, the LDLR is also modulated by PCSK9 or the inducible degrader of LDLR (IDOL) at post-transcriptional regulation [38, 39]. The PCSK9 and IDOL proteins decrease the LDLR abundance and reduce LDL uptake into liver cells. The extracellular mature PCSK9 protein has been demonstrated to bind the epidermal growth factor-like repeat A (EGF-A) domain of LDLR, which leads to internalization, impedes LDLR recycling and delivers LDLR to the lysosome for degradation [40]. The overexpression of PCSK9 or PCSK9-knockout mice shows an inverse relationship between LDLR and PCSK9 levels, indicating that PCSK9 protein also controls the plasma cholesterol homeostasis [41, 42]. Thus, we next investigated the effect of tanshinone IIA on PCSK9 expression in HepG2 cells. In the present study, we found that tanshinone IIA markedly inhibits the PCSK9 promoter activity and decreases the levels of PCSK9 mRNA and mature protein. Our current findings suggested that tanshinone IIA mediates the inhibition of PCSK9 expression, which leads to enhance the cell-surface LDLR and its LDL-uptake activity. PCSK9 inhibition therapy through monoclonal antibodies or small interfering RNA (siRNA) leads to higher levels of hepatic LDLR, lower circulating LDL-C levels, and improved cardiovascular disorder outcomes and does not show any adverse side-effects [43–45]. In the present study, the inhibition of PCSK9 gene expression by tanshinone IIA might be expected to serve as a novel approach to cholesterol management. In addition to PCSK9, E3-ubiqutin ligase IDOL protein has been reported to share the same LDLR substrate, although it has a distinct mechanism in modulating the LDLR abundance at the post-translational level [39]. In the present study, we found that the level of IDOL mRNA was decreased by approximately 35%, although the IDOL protein expression was slightly reduced in the tanshinone IIA (10 μM)-treated HepG2 cells (data not shown). The effect of tanshinone IIA on the IDOL-mediated LDLR modulation remains to be further investigated. Our current findings suggested that tanshinone IIA significantly increases the LDLR amount through the down-regulation of PCSK9 expression.

In the present study, we demonstrated that the PCSK9 mRNA is down-regulated by tanshinone IIA through the inhibition of promoter activity. The tanshinone IIA-responsive element was characterized and defined to the proximal promoter region (-411 to -336). Within this region of the PCSK9 promoter, there are three potential transcriptional factor binding sites, including the HNF-1α binding element [46], the sterol regulatory element for SREBP2 interaction [46] and FoxO3a binding site [15] that have been identified. We further analyzed the nuclear amounts of HNF-1α, SREBP2 and FoxO3a in tanshinone IIA-treated HepG2 cells. We found that tanshinone IIA (10 μM) slightly reduced nuclear SREBP2 level and dramatically enhanced the nuclear amount of FoxO3a in HepG2 cells. Our data revealed that FoxO3a plays a major role in the tanshinone IIA-mediated PCSK9 transcriptional suppression. The FoxO3a protein is reported to be an important transcription factor and is associated with several essential cellular functions including cell proliferation, apoptosis, metabolism, stress resistance and longevity in several cell types [47, 48].Tao et al. reported that FoxO3 overexpression improves hypercholesterolemia in obsess mouse models. The FoxO3 protein has been demonstrated to coordinate with NAD^+^-dependent deacetylase sirtuin 6 (Sirt6) for the suppression of SREBP2 expression and modulation of cholesterol biosynthesis in a mouse model [49]. In addition to regulate the SREBP2, FoxO3a has been reported to recruit Sirt6 to the proximal promoter region of PCSK9 and result in the deacetylation of histones H3 at lysine 9 (H3K9) and lysine 56 (H3K56), a repressed state of the chromatin of PCSK9 gene, to suppress the HNF-1α-mediated transcriptional activation, which leads to the down-regulation of PCSK9 mRNA expression [15]. In this study, we found that the accumulation of nuclear FoxO3a protein was markedly increased by tanshinone IIA in a dose-dependent manner in HepG2 cells. We demonstrated that tanshinone IIA increased the FoxO3a/PCSK9 promoter binding capacity in HepG2 cells. Moreover, we found that the level of nuclear HNF-1α protein was not altered, however, the PCSK9 promoter binding capacity of HNF-1α was significantly reduced in tanshinone IIA-treated cells. Our current findings supported that treatment of cells with tanshinone IIA causes an increase in nuclear FoxO3a protein accumulation may recruit Sirt6 deacetylase to promote histones modification, which leads to suppress the promoter-binding capacity of nuclear HNF-1α for PCSK9 transcription. Plas et al. reported that the FoxO3a is phosphorylated by protein kinase B (Akt) and is mainly retained in the cytoplasm for degradation, which decreases the transcriptional activity [50]. Nho et al. reported that the de-phosphorylation of FoxO3a by protein phosphatase-2A (PP2A) results in promoting its translocation to the nucleus [51]. Whether tanshinone IIA affects the kinase or phosphatase activity to increase the nuclear accumulation of FoxO3a protein needs to be further investigated.

Statins and ezetimibe are the most common and effective drugs for lowering plasma cholesterol levels and improving outcomes from coronary artery diseases [52–54]. Statins, 3-hydroxy-3-methylglutaryl-coenzyme A (HMG-CoA) reductase inhibitors, reduce cholesterol levels by inhibiting cholesterol biosynthesis. Statins have been reported to increase the LDLR mRNA level and to enhance the clearance of plasma cholesterol. However, statin therapy has some limitations including intolerance to statin treatment and increased plasma levels of PCSK9 [55, 56]. Statins increase PCSK9 as well as LDLR expression, which compromise the effect of the increased LDLR levels counteracting the ability to reduce the plasma LDL-cholesterol levels. In the present study, tanshinone IIA attenuated lovastatin- or simvastatin-induced PCSK9 mRNA and protein expression. Moreover, a combination of tanshinone IIA and lovastatin or simvastatin dramatically enhanced the LDL-uptake by HepG2 cells. Our results suggest that tanshinone IIA effectively reduces PCSK9 expression and could be used as an effective supplement to statin therapy for greater suppression of LDL-C.

In conclusion, we report that tanshinone IIA exerts the promising effect of PCSK9 down-regulation. Our data elucidates that tanshinone IIA mediates the suppression of PCSK9 in a FoxO3a-related manner, leading to an increase in LDL uptake in hepatic cells. Our findings provide a molecular basis of tanshinone IIA for the development of PCSK9 inhibitors for cholesterol homeostasis maintenance.
